# Miscellaneous Medical Intelligence

**Published:** 1851-03

**Authors:** 


### ARTICLE VII.

### MISCELLANEOUS MEDICAL INTELLIGENCE.

The distinguished Surgeon, B. W. Dudley, M. D., of Lexington, Kentucky, is afflicted with irritability of the eyes, which prevents a continuation of his Surgical Memoirs in the Transylvania Medical Journal.

There were 100 students in attendance upon the first course of lectures in the Kentucky School of Medicine.

There were 544 deaths from cholera in New Orleans, between November 1st and December 23, 1850, from which time it entirely disappeared.

From June to November, 1850 inclusive, there were 10,928 admissions into the Charity Hospital, New Orleans, and 818 deaths. A little less than eight per cent. of the deaths, 152, were from cholera.

The Editor of the American Journal of Dental Science, estimates that there are 6,600 ounces of gold used for filling teeth in the United States annually worth $198,000.

There were 202 students in attendance at the Cleveland Medical School the past winter, about 25 per cent less than last year. So far as we can learn there has been a great falling off of the number of students generally, probably owing to the influence of the California emigration.

Medical students have increased very much in Paris and London.

A boy nine years old, says the *Provincial Journal*, swallowed a silk handkerchief, a foot square, and passed it by the bowels three days after, perfect in every respect, except being soiled. The boy suffered no injury from being "wiped out.'

Dr. A. Stille, of Philadelphia, is travelling in Europe for his health. He has resigned the post of Secretary of American Medical Association, which he has filled with ability for several years. Dr. H. W. De Sausseur, of Charleston, S. C., is the Secretary, to whom all matters pertaining to the office should be directed.

Measels and hooping cough have been quite prevalent in Chicago during the winter just passed.

The demand for cod liver oil seems to be on the increase, indicating its more general employment.

There were about 1500 students in Philadelphia during the past winter and 700 in New York.

Professor White, of Buffalo, has gone to Europe on business connected with the Buffalo Medical College.

Professor Howard, of Columbus, Ohio, is also to start in a few days.

The Homœopaths have instituted a Hospital in London.

The cholera is reported to be prevailing on the Ohio River.

A census of the Physicians of Erie County, New York, has been taken by the County Medical Society; there are seventy-nine. Twenty are members of the Society. Six are reported graduates but not members. Ten are neither gradutes nor licentiares, but are respectable practitioners. Thirteen profess to be *regular* but are the veriest quacks. Four practice Homœopathy, Allopathy, or any thing else to get business. Two are "simon pure" Homœopaths. Four are Seroscopists. There are ignorant females pretending to general practice. Five Eclectics, and ten are Botanic, Thomsonian or Herb Doctors.

The *New York Register of Medicine and Pharmacy* publishes a table of the mortality of the City of New York for 1850, by which it appears there were 16,854 deaths. The largest number of deaths from any one disease was from consumption, it being 1801. The proportions from some of the other diseases is remarkable. It was from convulsions, 1280. From Inflammation of the Lungs, 922. From Dysentery, 786. From Dropsy in the Head, 729. From Marasmus, 729. From Cholera Infantum, 717. From Apoplexy, 561. And from Cholera, only 47. The number of deaths for 1847, was 15,-499; for 1848 it was 15,919, and for 1849, when the Cholera was epidemic, it was 23,785.

Mr. Haslem, of Boston, has invented and manufactured a new *vaginal speculum* made of glass, covered with silver on the outside and the silvering protected by a coat of gutta percha. Its advantage is that the mirror formed reflects the light better than any heretofore in use.

Mr. J. P. Cook has been appointed Professor of Chemistry in Harvard University in place of Professor Horsford.

The number of deaths in Boston in the year 1850, was 3,667.

